# Recent Advances in Imaging for Diagnosis, Monitoring, and Prognosis of Psoriatic Arthritis

**DOI:** 10.3389/fmed.2020.551684

**Published:** 2020-10-29

**Authors:** Angelo Fassio, Peter Matzneller, Luca Idolazzi

**Affiliations:** ^1^Rheumatology Unit, University of Verona, Verona, Italy; ^2^Rheumatology Service, South Tyrolean Health Trust, Silandro Hospital, Silandro, Italy

**Keywords:** psoriatic arthritis, psoriasis, biologics, magnetic resonace imaging (MRI), ultrasonography

## Abstract

Psoriatic arthritis (PsA) is an inflammatory condition characterized by a strong heterogeneity and multifaceted behavior. PsA manifests in two types—axial and peripheral—which may be present at the same time. Peripheral manifestations can be further divided into the articular (arthritis) and extra-articular (i.e., enthesitis and dactylitis) subgroups. In such a complex disease, imaging is often required to characterize the type of involvement and to evaluate the radiological damage and progression of PsA. In addition, imaging plays a pivotal role in clinical practice; that is, for axial involvement. Conventional radiology has been the main standard of reference for many years. However, in recent years, there has been growing interest in different imaging modalities, such as ultrasonography (US) and magnetic resonance imaging (MRI). All these techniques play a role in the diagnosis and follow-up of patients with PsA and cover all the types of the disease. US and MRI have good sensitivities and specificities for detecting synovitis, and this may be helpful for differential diagnosis with other musculoskeletal diseases and useful in the early or preclinical phases of the disease. However, US is not useful in the diagnosis of axial PsA. In addition, other modalities have been investigated in the field of PsA imaging. Computed tomography (CT), in particular, dual energy-CT and high-resolution peripheral CT (HRpQ-CT) might play an important role in the assessment of bone damage, erosions, and new bone formation. Regarding advanced functional imaging, FDG PET/CT is another interesting technique for exploring disease activity.

## Introduction

Psoriatic arthritis (PsA) is characterized by a wide spectrum of features and can express itself as part of the psoriatic disease (1). Starting from the early phase of the disease, imaging plays a crucial role for diagnosis, prognosis, and management. The main types of PsA can be distinguished in the axial and peripheral regions, and they can be present alone or concurrently. Furthermore, peripheral PsA may involve joints as well as extra-articular structures, such as the entheses (2), and a concomitant inflammation of these can occur, such as in dactylitis (3). Patients with PsA often need a comprehensive evaluation that involves several imaging techniques. Clinicians should carefully choose proper imaging techniques in favor of achieving early diagnosis and, consequently, favorable clinical outcomes. This is especially true for axial involvement (4), for which under-diagnosis is still an issue (5). The definition of the type(s) and extension of the involvement is currently a challenging aspect of imaging in rheumatology because the different spondyloarthritides (SpA) forms have various clinical presentations (6, 7).

Given the heterogeneous nature of PsA, rheumatologists often face presentations of the disease that differ significantly between cases. Imaging can differentiate between PsA types as well as highlight differences to other SpA (i.e., ankylosing spondylitis).

The radiographic signs of structural axial damage in SpA typically involve erosive changes, followed by new bone formation and ankylosing alterations (8). The assessment of these changes is useful for both management and prognosis. In particular, disease activity is a strong determinant for the treatment choices of rheumatologists. Magnetic resonance (MRI) and ultrasonography (US) are helpful in the objective reflection of disease activity, and conventional radiography (XR) and especially computed tomography (CT) are extremely useful in the detection and monitoring of structural pathological changes (9). Recent studies have highlighted the close relationship between bone alterations and bone markers (10, 11). Cytokine dysregulation in SpA has been associated with the development of pathological bone alterations (12), suggesting that, in the future, the appropriate drug choice might be guided by the main cytokine axis involved (10, 13). However, it is currently not known how to assess and interpret the cytokine profile in clinical practice. On the other hand, imaging can already help in defining a precision medicine approach.

**Figure 1:**
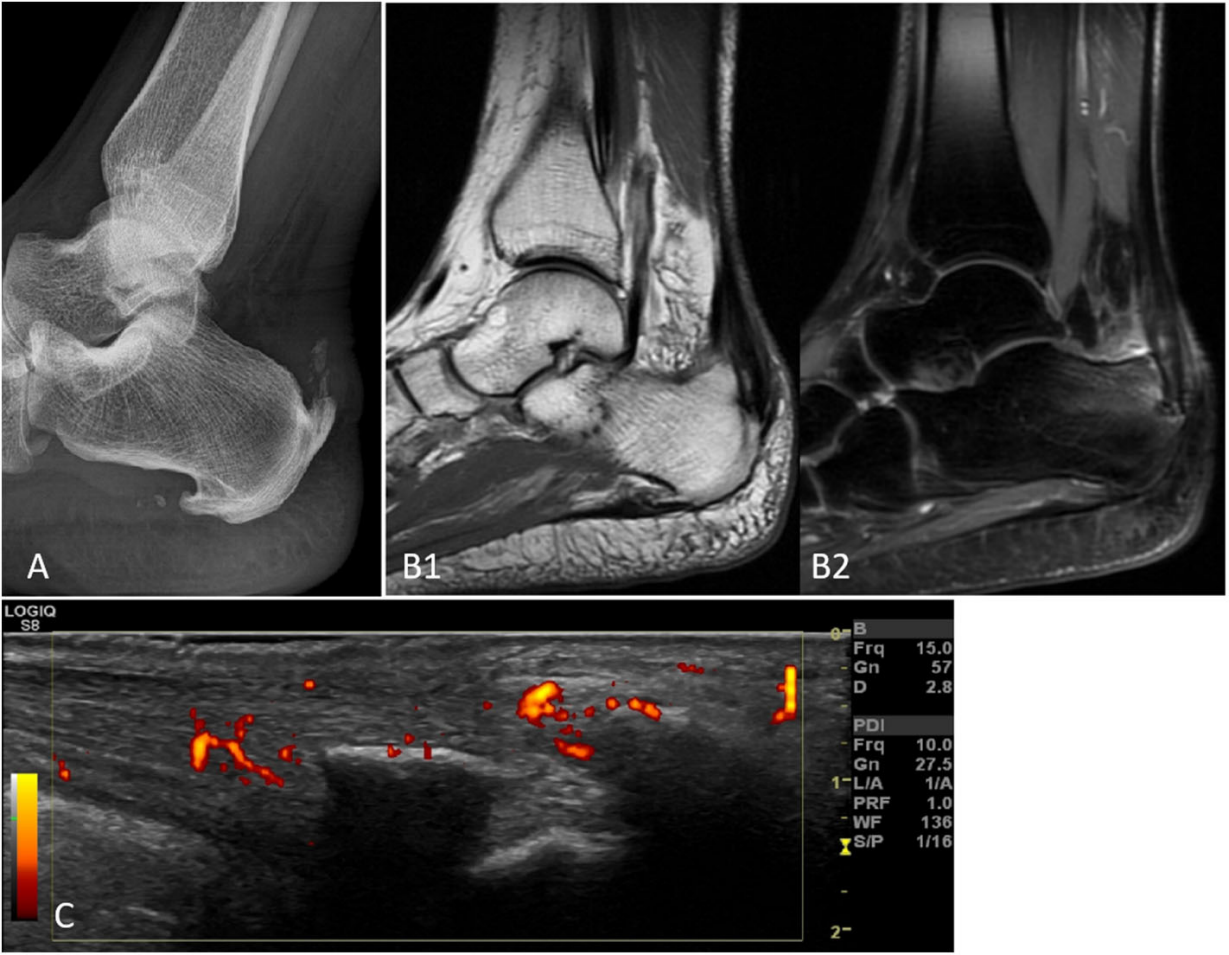
Achilles' tendon enthesitis of the same patients with peripheral SpA as assessed by XR **(A)**, MRI with T1 sequences **(B1)**, MRI with contrast-enhanced sequences **(B2)**, and PD US **(C)**. The various imaging techniques differently emphasize the pathological changes related to the disease, i.e., mainly bone changes with XR and soft tissue changes with MRI and US. Only MRI (B2) can document subcortical osteitis/bone marrow edema. Abbreviations: MRI, magnetic resonance imaging; SpA, spondyloarthritis; US, ultrasonography.

This review aims to discuss the role of imaging in the diagnosis, management, and prognosis of PsA over the last 5 years, including fundamental papers from inception to 2020. We used the MEDLINE/PubMed, CINAHL, EMBASE, and Cochrane Library databases. In addition, we manually searched references from the retrieved articles and explored a number of related websites. It covers the axial and peripheral types of the disease, and the imaging techniques taken into account are MRI, US, CT, and XR as well as novel techniques of advanced imaging, such as high-resolution peripheral computed tomography (HRpQ-CT) and dual energy computed tomography (DECT). Figure 1 depicts an Achilles' tendon enthesitis assessed with different modalities.

## XR

Regarding XR, several papers were published between 2016 and 2019, most of which are related to therapy response. XR maintains an important role in the study of SpA and PsA because it is the discriminant for radiographic and nonradiographic types of sacroiliitis, but also because it is very helpful in differential diagnosis with other diseases, such as osteitis condensans.

Among the studies on diagnosis and prognosis, the paper by Aydin et al. (4) is focused on the impact and outcomes of undiagnosed axial PsA. Starting from PsA patients in the Turkish National Registry, 1195 were recruited, 112 of which fulfilled the modified New York (NY) criteria, even if they had not received a formal diagnosis of the axial disease. This group of patients also showed a worse outcome on peripheral PsA with a higher number of tender and swollen joints, but similar patients reported outcomes.

Salaffi et al. (14) propose a novel score for the staging of structural damage using XR, named the Simplified Psoriatic Arthritis Radiographic Score (SPARS). The scoring system considers the combination of erosions, joint-space narrowing, and bone proliferation. The method is compared with the PsA Ratingen score (PARS) (15) and the modified Sharp/Van der Heijde score (16). Regarding convergent validity, SPARS shows a strong correlation with both of them, with *r* = 0.904 and 0.926 (*p* < 0.0001), respectively. SPARS is advantageous in that it is time saving and has a good agreement between readers.

Regarding treatment, Allar et al. (17) explore the radiographic change, its evolution, and the effects of transitioning from conventional DMARD (cDMARD) to biological DMARD (bDMARD). A relatively small cohort (*N* = 28) was retrospectively conducted. The cohort fulfilled the CASPAR criteria and belonged to the Bath longitudinal cohort. Data analysis considered three time points: at baseline, at first prescription of bDMARD, and 5 years after the start of tumor necrosis factor-α inhibitors (TNFi). From baseline to commencing bDMARD, the rate of erosion progression was 2.1 (IQR, 0.88–3.93). The study ran for 5 years after commencing the first bDMARD, and the rate of erosion changed to 0.0 (IQR = 0.00–0.81), suggesting a slowing down of the process due to this intervention.

A subanalysis of GO-REVEAL data by Kerschbaumer et al. (18) concludes that the responsiveness of a functional limitation decreases with increasing joint damage in a cohort of patients treated with golimumab, which were divided into two groups (remission and responders with previous functional impairment). The increase in the total modified Sharp/Van der Heijde score results was strongly associated with higher values of Health Assessment Questionnaire (HAQ), for both joint space narrowing and erosions, but the latter was less determinant for disability even though the comparison with the remission group showed a strong significance (*p* < 0.0001). Data from the FUTURE5 study (19) show a significant inhibition of the modified Sharp/Van der Heijde score at week 24 in any group, disregarding drug dosage. Similar data were published for FUTURE1 (20), with a rate of nonprogressors equal to 78% for naïve to TNFi patients and 65.5% for the group that previously experienced therapy with TNFi. Another subanalysis from the GO-VIBRANT study (21) highlights the inhibition of the progression in structural damage in a cohort of patients treated with intravenous golimumab 2 mg/kg. The PsA-modified Sharp/Van der Heijde score shows a *p* < 0.001 in every subanalysis, for example, regarding erosions and joint space narrowing, demographic data, baseline characteristics, and concomitant treatment. Data suggest the beneficial effects of golimumab not only for the progression, but also for the prevention of new joint erosion, with a rate of no new joint erosions of 74.3 vs. 54% in the placebo group (*p* < 0.001).

## US

US is a safe, readily available, and inexpensive instrument to study articular and extra-articular pathologies in patients with rheumatic diseases. The increasing role of musculoskeletal US in the diagnosis and management of PsA is reflected by the 2015 EULAR recommendations on the use of imaging in SpA (22). However, the optimal use of this technique in the clinical management of PsA is still to be defined, and data in this regard are lacking. This section constitutes an attempt to resume the most relevant publications addressing this issue in the last 3 years.

As far as the diagnosis of PsA is concerned, a higher sensitivity of US in detecting synovitis compared to clinical examination has been reproducibly confirmed by several controlled studies in the past. However, the value of US in addition to clinical examination (compared to clinical examination alone) in making the diagnosis of PsA is yet to be assessed (23).

Recently, in a study on patients with PsA and diagnosed according to the CASPAR criteria, US was able to detect markedly more joints with synovial proliferation (108 out of 840 joints) compared to clinical examination (32 and 59 of 840 joints graded as swollen and tender, respectively). In addition, the authors argue that US offers the opportunity to discriminate arthritis from tenosynovitis and subcutaneous edema while physical examination cannot distinguish between these different features (24).

In the differential diagnosis of early PsA and seronegative RA, it might be useful to combine US and dermoscopy. Detection of at least one extra-synovial feature using hand US and dotted vessels using nailfold dermoscopy was shown to be significantly associated with early PsA (25).

US could also help to identify patients with psoriatic arthralgia, that is, psoriasis (PSO) associated with nonspecific joint symptoms, who may be at risk of developing PsA. These patients were shown to have US signs of tenosynovitis significantly more often than patients with PSO alone (26).

Similarly, the presence of soft tissue edema, one of the anatomical components of dactylitis in PsA, might not only be useful as a diagnostic element in order to differentiate PsA from RA but also serve as an indicator of the disease severity. A novel US definition, including a grayscale and power Doppler (PD) scoring system of soft tissue edema in PsA, was proposed recently (26).

Another noteworthy finding in a study by Najm et al. (27) shows a good correlation of the degree of PD in US-detected knee joint synovitis with histopathological inflammation. The only other study that previously attempted to show such an agreement between US and a histopathological gold standard failed (28).

In a prospective observational study of 228 patients with recent-onset arthritis of the hands, thickening of the flexor tendon pulleys was reported to be a sensitive (80%) albeit unspecific (71%) US sign of PsA, which may, thus, be helpful in the diagnostic process if considered alongside other criteria (29). The same thickening of the flexor tendon pulleys, interpreted as “Deep Koebner's” response to physical stress, was found significantly more often in patients with established PsA than in patients with PSO alone, rheumatoid arthritis, or healthy controls (30).

US of the nail unit has drawn a great deal of attention in recent years. Nail involvement is a hallmark of psoriatic disease and is known to be associated with the development of PsA (31). The loss of the trilaminar structure of the nail plate, involvement of the extensor tendon at the level of the distal interphalangeal joint, and increased vascularization are the main features of psoriatic onychopathy. However, hypervascularization of the nail bed may also be present in healthy subjects (32). Similarly, thickening of the nail plate is also described in patients with osteoarthritis, whereas PD signal at entheseal levels seems to be quite specific for PsA. Overall, these US-described alterations of the nail unit are reported with a highly variable prevalence in the literature (33). Hence, despite promising clues, further research and standardization of the technique are required in order to reliably use nail US for diagnosis, prognosis, and monitoring of PsA.

US may also serve as an instrument to monitor disease activity and/or response to therapy.

A longitudinal study by Pukšić et al. (34) shows only poor a correlation between disease remission determined by the Disease Activity for Psoriatic Arthritis (DAPSA) score and PD US, indicating that the two measures depict different aspects of the disease. Accordingly, objective markers of inflammation, such as the 66 swollen joint count, CRP, and ESR, showed longitudinal agreement with PD US, whereas no correlation was observed for the pain-related components of DAPSA as well as patient-reported outcome measures (PROMs).

Similarly, pain and pain-related items were the main reasons why PsA patients with the absence of US signs of inflammation were classified with higher disease activity in another study comparing clinical and US definitions of low disease activity (LDA) (35). Both studies suggest that US may be helpful in objectively assessing residual inflammation, especially in patients with high, pain-driven, clinical scores but low clinical evidence of inflammation.

A study investigating PsA patients under IL-17 inhibition with secukinumab showed a significant decrease in PD US signs of joint inflammation after 24 weeks of treatment in good agreement with equally significant changes in MRI-detected synovitis as well as clinical measures of joint diseases such as DAPSA and DAS28-ESR (36).

In a longitudinal study on patients with clinically inactive juvenile idiopathic arthritis, US joint abnormalities, such as synovial hyperplasia, joint effusion, and PD signal, were shown to be strong predictors of the individual relapse risk (37).

In general, despite increasing data on the optimal integration of US into clinical practice, some areas remain controversial and/or fragmentary, particularly when it comes to the identification of US findings with potential prognostic value. Important answers in this regard are expected from future studies.

A brief summary of the main studies is reported in Table 1.

**Table 1:** Revised articles about US.

**Ultrasonography and diagnosis in PsA**			
**First Author**	**Reference**	**Title**	**Description**
Zabotti et al. (26)	*RMD Open*. (2019)	Transition phase toward psoriatic arthritis: clinical and ultrasonographic characterization of psoriatic arthralgia.	Tenosynovitis was associated with arthralgia in subjects with psoriasis. Baseline US evidence of enthesitis was associated with clinical PsA development in the longitudinal analysis.
Idolazzi et al. (38)	*Clin Rheumatol*. (2019)	The ultrasonographic study of the nail reveals differences in patients affected by inflammatory and degenerative conditions.	Ultrasonography nails in psoriasis/PsA/osteoarthritis
Tinazzi et al. (39)	*Med Ultrason*. (2019)	Ultrasonographic detection, definition and quantification of soft tissue oedema in psoriatic dactylitis.	Soft tissue edema in psoriatic dactylitis
Tang et al. (40)	*Quant Imaging Med Surg*. (2020)	Ultrasound assessment in psoriatic arthritis (PsA) and psoriasis vulgaris (non-PsA): which sites are most commonly involved and what features are more important in PsA?	Affected sites compared between PsA and PsO patients
Helliwell et al. (41)	*J Rheumatol*. (2019)	Comparing psoriatic arthritis low-field magnetic resonance imaging, ultrasound, and clinical outcomes: data from the TICOPA trial.	Comparison MRI and US
Florescu et al. (24)	*Curr Health Sci J*. (2019)	The Role of Ultrasound in Assessing Hand Joints and Tendons in Psoriatic Arthritis.	Most affected sites: 3rd finger, flexor tendons, extensor carpi ulnaris, flexor carpi radialis
Furlan et al. (29)	*J Ultrasound*. (2018)	The thickening of flexor tendons pulleys: a useful ultrasonographical sign in the diagnosis of psoriatic arthritis.	Good sensitivity, poor specificity
Krajewska-Włodarczyk et al. (42)	*Biomed Res Int*. (2018)	Ultrasound assessment of changes in nails in psoriasis and psoriatic arthritis.	The findings of this study may indicate an association of an inflammation in the nail bed with PsA development. Apart from a direct assessment of the described morphological changes of nails, a US examination could prove useful in an assessment of intensity of a local inflammation as a prognostic factor for PsA development.
Mondal et al. (43)	*Rheumatol Int*. (2018)	Assessment of nail unit structures by ultrasound in patients with psoriatic arthritis and their correlations with disease activity indices: a case-control study.	Ultrasound (USG) of nail was performed to assess, (1) morphological alterations of nail plates in psoriatic arthritis (PsA) patients, (2) differences of nail unit parameters [nail bed thickness (NBT), nail matrix thickness (NMT) and nail plate distance (NPD)] in PsA patients from healthy controls (3) correlation of nail unit parameters with PsA disease activity indices. Total of 895 fingernails (448 nails of 45 PsA patients and 447 of 45 controls) were evaluated by USG. Psoriasis Area and Severity Index (PASI), Disease Activity in Psoriatic Arthritis (DAPSA), and Nail Psoriasis Severity Index (NAPSI) were calculated in PsA patients. Nail unit parameters were compared between two study groups. Correlation study was done between nail unit parameters and disease activity indices. All PsA patients showed ultrasound evidence of nail plate changes (87.95% of the total fingernails and 75.34% of the clinically normal nails). Loosening of the ventral nail plate border was most common (51.79%). Mean NBT (PsA: 0.19 ± 0.035 cm, control: 0.17 ± 0.018 cm, *p* = 0.003) and mean NMT (PsA: 0.32 ± 0.041 cm, control: 0.28 ± 0.031 cm, *p* ≤ 0.0001) were significantly increased in the PsA patients. Moderately positive correlation was observed between NAPSI score and mean NMT (Spearman *r* = 0.411, 95% confidence interval: 0.125–0.634, *p* = 0.005). USG evidence of nail plate alterations was frequent among PsA patients, even in clinically normal nails. Increased mean nail bed and matrix thickness were noted in PsA patients. Mean NMT had a moderately positive correlation with NAPSI score.
Macía-Villa et al. (44)	*Clin Exp Rheumatol*. (2018)	What is metacarpophalangeal joint swelling in psoriatic arthritis? Ultrasound findings and reliability assessment.	Clinical swelling was present in 60 joints whereas US detected IAS and/or PTI in 75 MCPj. GS PTI in at least one MCPj was found in 19 patients and 41 joints, concurring with clinical swelling in 30/41. GS IAS in at least one MCPj was found in 23 patients and 63 joints, concurring with clinical swelling in 37/63. The inter-reader reliability was good for PD PTI and moderate for GS PTI.
Højgaard et al. (45)	*Arthritis Care Res (Hoboken)*. (2019)	Pain mechanisms and ultrasonic inflammatory activity as prognostic factors in patients with psoriatic arthritis: a prospective cohort study.	More than one-third of patients with PsA presented with WP, which was associated with worse patient-reported scores and failure to achieve minimal disease activity following conventional synthetic or biologic disease-modifying antirheumatic drug therapy. PsA activity by color Doppler US had no influence on subsequent treatment response in this PsA cohort.
Idolazzi et al. (46)	*Med Ultrason*. (2018)	Ultrasonography of the nail unit reveals quantitative and qualitative alterations in patients with psoriasis and psoriatic arthritis.	Multivariate analysis of variance was performed between groups. *Post*-*hoc* analysis underlined the differences between healthy and affected regarding nail plate thickness (0.063 ± 0.011 cm for patients with psoriasis, 0.065 ± 0.014 cm for patients with psoriatic arthritis and 0.051 ± 0.006 cm for healthy controls, *p* < 0.05). Elementary lesions of nail plate and nail bed were compared using Pearson's chi square test between patients in psoriasis and psoriatic arthritis groups, with no differences except for a trend for onycholisis and crumbling (*p* = 0.07 and 0.06, respectively) in the psoriatic arthritis group. ROC curves were calculated (AUC = 0.68) obtaining also quantitative cut offs for nail plate and nail bed thickness in the affected vs. healthy patients.
Zabotti et al. (25)	*Ann Rheum Dis*. (2018)	Ultrasonography in psoriatic arthritis: which sites should we scan?	In psoriatic arthritis (PsA), ultrasonography (US) plays a growing role in the differential diagnosis and in monitoring treatment response (1), PsA is a heterogeneous disease with different domains and peculiar sites involved (2). Therefore, a dedicated US composite score is needed to monitor disease activity and to identify structural damage progression. A recently published Systematic Literature Review (SLR) identified only two US scores specifically developed for PsA (i.e., 5TPD and PsA-Son) and, although these had a good sensitivity to detect inflammation and a good feasibility, they have not been validated in other series (1, 3, 4). Recently, the Study Group for US of the Italian Society of Rheumatology promoted the Ultrasound in PSoriatic Arthritis TREAtMent (UPSTREAM) study (registered at ClinicalTrial.gov, NCT03330769). UPSTREAM is a multicenter observational prospective cohort study and it represents the first
Tinazzi et al. (30)	*Ann Rheum Dis*. (2018)	“Deep Koebner” phenomenon of the flexor tendon-associated accessory pulleys as a novel factor in tenosynovitis and dactylitis in psoriatic arthritis.	In established PsA, the accessory pulleys are thickened compared with RA, PsO, or HCs and especially in subjects with a history of dactylitis. These findings implicate the involvement of pulleys in PsA-related tenosynovitis and dactylitis supporting the idea of deep koebnerization in dactylitis and sites of high physical stress.
Zabotti et al. (25)	*J Rheumatol*. (2018)	Early psoriatic arthritis vs. early seronegative rheumatoid arthritis: role of dermoscopy combined with ultrasonography for differential diagnosis.	Integrated rheumatological-dermatological clinical evaluation may be helpful in identifying patients with EPsA misclassified as seronegative ERA. Additionally, US and dermoscopy may be used as supportive tools in identifying subclinical psoriatic features, which may come in handy in distinguishing EPsA from ERA.
Ahmed et al. (47)	*Open Access Maced J Med Sci*. (2017)	Ultrasonographic enthesopathy and disease activity in psoriatic arthritis.	Of 70 entheses in 35 active PsA patients, the most entheseal abnormalities were tender plantar fascia (18.5%), tender Achilles tendon (37.8%). PASDAS was a direct highly significant correlated with plantar fascia and Achilles tendon thickness in in active PsA (*r* = 0.823 and 0.796, *p* < 0.001, respectively). Musculoskeletal US is an accurate and low-cost method for assessment of enthesopathy with significant correlation to disease activities in psoriatic arthritis.
Aydin et al. (32)	*Clin Exp Rheumatol*. (2017)	Vascularity of nail bed by ultrasound to discriminate psoriasis, psoriatic arthritis and healthy controls.	
Uson et al. (48)	*Reumatol Clin*. (2018)	Recommendations for the use of ultrasound and magnetic resonance in patients with spondyloarthritis, including psoriatic arthritis, and patients with juvenile idiopathic arthritis.	
Fiocco et al. (49)	*Clin Rheumatol*. (2017)	Quantitative imaging by pixel-based contrast-enhanced ultrasound reveals a linear relationship between synovial vascular perfusion and the recruitment of pathogenic IL-17A-F^+^IL-23^+^ CD161^+^ CD4^+^ T helper cells in psoriatic arthritis joints.	To develop quantitative imaging biomarkers of synovial tissue perfusion by pixel-based contrast-enhanced ultrasound (CEUS), we studied the relationship between CEUS synovial vascular perfusion and the frequencies of pathogenic T helper (Th)-17 cells in psoriatic arthritis (PsA) joints. Eight consecutive patients with PsA were enrolled in this study. Gray scale CEUS evaluation was performed on the same joint immediately after joint aspiration, by automatic assessment perfusion data, using a new quantification approach of pixel-based analysis and the gamma-variate model. The set of perfusional parameters considered by the time intensity curve includes the maximum value (peak) of the signal intensity curve, the blood volume index or area under the curve, (BVI, AUC) and the contrast mean transit time (MTT). The direct ex vivo analysis of the frequencies of SF IL17A-F^+^CD161^+^IL23^+^ CD4^+^ T cells subsets were quantified by fluorescence-activated cell sorter (FACS). In cross-sectional analyses, when tested for multiple comparison setting, a false discovery rate at 10%, a common pattern of correlations between CEUS Peak, AUC (BVI), and MTT parameters with the IL17A-F^+^IL23^+^–IL17A-F^+^CD161^+^–and IL17A-F^+^CD161^+^IL23^+^ CD4^+^ T cells subsets, as well as lack of correlation between both peak and AUC values and both CD4^+^T and CD4^+^IL23^+^ T cells, was observed. The pixel-based CEUS assessment is a truly measure synovial inflammation, as a useful tool to develop quantitative imaging biomarker for monitoring target therapeutics in PsA.
**Ultrasonography and monitoring in PsA**			
Ceccarelli et al. (50)	*Clin Rheumatol*. (2019)	Musculoskeletal ultrasound in monitoring response to apremilast in psoriatic arthritis patients: results from a longitudinal study.	MSUS can monitor articular and periarticular response to apremilast in PsA
Bosch et al. (35)	*Rheumatology (Oxford)*. (2019)	Evaluating current definitions of low disease activity in psoriatic arthritis using ultrasound.	The LDA cut-offs of DAPSA, PASDAS, Composite Psoriatic Disease Activity Index, minimal disease activity, but not DAS28-CRP are capable of distinguishing between high and low ultrasound activity. Pain and pain-related items are the main reason why PsA patients without signs of ultrasound inflammation are classified with higher disease activity.
Pukšić et al. (34)	*RMD Open*. (2018)	DAPSA and ultrasound show different perspectives of psoriatic arthritis disease activity: results from a 12-month longitudinal observational study in patients starting treatment with biological disease-modifying antirheumatic drugs.	DAPSA was not associated with US inflammatory findings which indicates that DAPSA and US may assess different aspects of PsA activity.
Alivernini et al. (51)	*Ann Rheum Dis*. (2017)	Synovial features of patients with rheumatoid arthritis and psoriatic arthritis in clinical and ultrasound remission differ under anti-TNF therapy: a clue to interpret different chances of relapse after clinical remission?	PDUS-negative patients with RA in remission have comparable synovial histological features than PDUS-negative patients with RA in LDA. However, patients with PsA in remission are characterized by a higher degree of residual synovial inflammation than patients with RA in remission, despite PDUS negativity under TNF inhibition.
Ramírez et al. (52)	*Clin Exp Rheumatol*. (2017)	Differing local and systemic inflammatory burden in polyarticular psoriatic arthritis and rheumatoid arthritis patients on anti-TNF treatment in clinical remission.	Polyarticular PsA patients in remission had lower levels of local (US synovitis) and systemic inflammation than RA patients in remission, even though a significantly higher percentage of PsA patients were on tapered doses of anti-TNF, mainly in monotherapy.
Kampylafka et al. (36)	*Arthritis Res Ther*. (2018)	Resolution of synovitis and arrest of catabolic and anabolic bone changes in patients with psoriatic arthritis by IL-17A blockade with secukinumab: results from the prospective PSARTROS study.	Treatment with secukinumab led to significant improvement of signs and symptoms of PsA; 46% reached MDA and 52% DAPSA low disease activity. MRI synovitis (*P* = 0.034) and signal in PDUS (*P* = 0.030) significantly decreased after 24 weeks of treatment. Bone erosions in MRI and HR-pQCT and enthesiophytes in the HR-pQCT did not show any progression, and structural integrity and functional bone strength remained stable.

## MRI in Peripheral Arthritis

MRI is a useful and sensitive tool to investigate the anatomical structures involved in peripheral arthritides. The 2015 EULAR recommendations for the use of imaging in SpA considers MRI as a valuable tool for supporting the diagnosis and monitoring disease activity and structural damage in peripheral SpA (pSpA) (22). However, the available data are still relatively scarce, and much of our knowledge is derived from studies on RA.

Typical MRI findings in peripheral PsA include synovitis, tenosynovitis, periarticular inflammation, bone edema, erosions, and bone proliferation (53). All these features have been included in a scoring system (PsAMRIS) (53), which was validated in 2015 with good intrareader agreement and sensitivity to change (54). Currently, MRI is still the only validated tool for the joint assessment of PsA (55). In addition, very recently, OMERACT has proposed the definitions for a bone and soft-tissue-based scoring system for enthesitis using the heel as a model (HEMRIS) (56) with good reliability values.

As is known, MRI scanning protocols for the assessment of peripheral PsA do not differ significantly from RA protocols and recommend the adoption of coronal and sagittal T1- and T2-weighted images with fat suppression or STIR/TIRM (53, 57). Acquisition of additional T1-weighted sequences after intravenous gadolinium-containing contrast agent is needed for optimal assessment of synovitis, osteitis, and tenosynovitis (53, 57) because fat-suppressed sequences alone may not be able to discriminate between joint fluid and an inflamed (and, therefore, highly vascularized) synovium.

As already discussed, enthesitis is one of the hallmarks of PsA, and MRI has been shown to be sensitive in detecting structural changes of this kind (58). Narváez et al. (59) investigate the usefulness of MRI as a complementary approach for differential diagnosis in ambiguous cases in a cohort of 20 patients with early RA and 17 with early PsA. No significant differences were found in joint synovitis, and tenosynovitis of the flexor tendons appeared to be more often present in PsA (70.6 vs. 30%, *p* = 0.014, with a sensitivity and specificity for PsA of 71 and 70%, respectively) and tenosynovitis of the extensors (at the wrist) in RA (70 vs. 29.6%, *p* = 0.014, with a sensitivity and specificity of 70 and 75%, respectively). Interestingly, the best values of specificity for PsA (100%) were observed for the findings of enthesitis and diaphyseal bone edema. In addition, soft tissue edema was a highly specific finding for PsA (95% specificity). Indeed, extracapsular involvement has often been observed in other small studies (60–62). Overall, an imbalance in the prevalence of this feature between PsA and RA has been consistently shown, yet the data are currently insufficient to recommend the adoption of these features as a criterion for differential diagnosis in clinical practice.

In 2019, the results of a sub-study of the TICOPA trial were published (41). The study evaluated the performance of noncontrast MRI (single hand, assessed by PsAMRIS) with an additional global inflammation score. US (same hand) was also performed. Good interobserver reliability was confirmed for all the features analyzed (synovitis, flexor tenosynovitis, periarticular inflammation, bone erosion, and bone edema) except for bone proliferation, which showed poor measures or inter-observer intra-class correlation. Overall, both the PsAMRIS and US inflammation scores demonstrated good responsiveness, and a good correlation between baseline and change scores for MRI and US was found as well as a good correlation between baseline MRI imaging and clinical scores. The authors also concluded that, despite the limitations of the study (i.e., low-field, single-region examination), the results showed a good sensitivity to change and, therefore, supported the construct validity of the change scores.

The finding of low inter-reliability values for bone proliferation is not unexpected. Pathological bone proliferation is typical of spondyloarthropathies and PsA (13, 36) although it is not a common finding in RA. Given that much of our knowledge on structural damage as well as the tools adopted for its assessment have been readapted from RA, anabolic changes represent a relatively new feature to investigate. However, interest is growing, and MRI offers intriguing insights on the matter. In the PsARTROS study (36), an open-label single group study on 20 patients affected by PsA and receiving treatment with secukinumab for 24 weeks, PsAMRIS score (along with other imaging techniques) of the hands was performed to assess synovitis, periarticular inflammation, bone erosion, and enthesophyte formation. Even in this relatively small sample, MRI proved to be effective in showing therapeutic response in terms of synovitis. Periarticular inflammation, which, in part, resembles enthesitis, completely resolved upon secukinumab treatment. In addition, both MRI and HRpQ-CT showed the absence of the development of both erosive and bone anabolic damage, supporting the possibility of the arrest of progression of anabolic changes in PsA with secukinumab.

Another relevant field of research is the characterization of subclinical joint and tendon involvement in patients diagnosed with cutaneous PSO. In 1998, Offidani et al. (63) observed that, in a cohort of 25 patients free of joint symptoms, 68% of them were positive for one or more arthritic signs on MRI with soft-tissue abnormalities being the most prevalent (44%). Similar observations have been repeatedly published in the literature (64–67) with quite a heterogeneous prevalence depending on the sites investigated and the described lesions. Overall, the current evidence seems to suggest that more than half of the otherwise asymptomatic subjects affected by PSO show a subclinical inflammatory involvement. On this account, the data from a prospective single-arm open-label study on 20 patients with PSO without an established diagnosis of PsA treated with secukinumab (an anti-interleukin-17 monoclonal antibody) have been recently published. The cohort concerned patients at high risk for developing overt arthritic involvement (85% of them reported arthralgia, and 40% had tender joints at baseline examination, and 83% had at least one inflammatory lesion on MRI). The extent of arthralgia significantly declined after 24 weeks of secukinumab treatment, and a significant improvement in patient-reported outcomes and quality of life was observed. In addition, the sequential assessment of joint inflammation by MRI showed a significant decrease in global PsAMRIS and PsAMRIS synovitis scores. The possibility of identifying patients in a prodromal phase for the development of symptomatic joint involvement and to successfully intervene with a specific treatment is intriguing. In particular, we consider the benefits of an early treat-to-target approach in PsA (68).

## MRI and Axial Involvement

As was the case in peripheral PsA, in axial PsA (ax-PsA), most of our imaging knowledge is derived from a wider population. In this case, it is carried out from studies involving patients affected by SpA, and only a little research has been conducted specifically on ax-PsA. This strategy, unavoidable in a setting characterized by the scarcity of data, is affected by some limitations. Indeed, it is known that although the inflammatory ax-PsA can be indistinguishable from other forms of axial SpA, such as ankylosing spondylitis (AS), it can also differ from it in several respects: Overall, the available literature seems to suggest clinically, radiographically, and prognostically worse axial disease in patients with AS than in patients with ax-PsA (7).

Nevertheless, MRI acquisition protocols and definitions for active and structural lesions are also generally used for ax-PsA (69).

In particular, the correlation between the clinical diagnosis of inflammatory back pain and its relative MRI findings is one of the most important topics to clarify. Monaldo-Ficco et al. (70) report, in a cohort of 125 patients (including both peripheral and/or ax-PsA), that, in less than half of the subjects with a clinical diagnosis of inflammatory back pain, structural and/or inflammatory lesions were observed, and ~30% had degenerative changes. On the other hand, of the 46 patients with mechanical back pain, 20% had changes consistent with spondylitis, whereas 72% had only degenerative changes. The authors conclude that inflammatory back pain might result from mechanisms other than inflammation. In addition, we should also suspect that symptoms of ax-PsA might not present as classical inflammatory low back pain in a nonnegligible proportion of patients.

Given the somewhat unpredictable distribution of peripheral, axial, and soft-tissue involvement in PsA, whole-body MRI has been investigated and has been shown to allow effective simultaneous assessment of peripheral and axial joints in PsA (71). However, this head-to-toe technique is affected by practical limitations and requires further testing before its consideration for a wider setting.

A brief summary of the main studies is reported in Table 2, divided according to the different types.

**Table 2:** Revised articles about MRI, split for peripheral, or axial subset of disease.

**MRI and peripheral disease**			
**First Author**	**Reference**	**Title**	**Description**
Glinatsi et al. (54)	*J Rheumatol*. (2015)	Validation of the OMERACT psoriatic arthritis magnetic resonance imaging score (PsAMRIS) for the Hand and foot in a randomized placebo-controlled trial	PsAMRIS showed overall good intrareader agreement in the hand and foot, and inflammatory feature scores were responsive to change, suggesting that PsAMRIS may be a valid tool for MRI assessment of hands and feet in PsA clinical trials.
Mathew et al. (56)	*J Rheumatol*. (2019)	The OMERACT MRI in enthesitis initiative: definitions of key pathologies, suggested MRI sequences, and a novel heel enthesitis scoring system	Heel Enthesitis Scoring System (HEMRIS) showed to be reliable among trained readers and promising for clinical trials.
Feydy et al. (58)	*Ann Rheum Dis*. (2012)	Comparative study of MRI and power Doppler ultrasonography of the heel in patients with spondyloarthritis with and without heel pain and in controls	Heel MRI and PDUS frequently show inflammatory lesions in SpA, particularly in painful heels. However, they were also often abnormal in controls. These results suggest that heel MRI and PDUS cannot be used for the diagnosis of SpA. However, PDUS and MRI may be useful for the depiction and assessment of enthesis inflammatory lesions in patients with SpA with heel pain.
Narvàez et al. (59)	*Semin Arthritis Rheum*. (2012)	Can magnetic resonance imaging of the hand and wrist differentiate between rheumatoid arthritis and psoriatic arthritis in the early stages of the disease?	Significant differences were observed in the MRI findings of the hand and wrist that can help to distinguish between RA and PsA in the early stages of disease. This imaging method was suggested to be able to help in the differential diagnostic process in selected patients in whom diagnosis cannot be unequivocally established after conventional clinical, biochemical, and radiographic examinations.
Helliwell et al. (41)	*J Rheumatol*. (2019)	Comparing psoriatic arthritis low-field magnetic resonance imaging, ultrasound and clinical outcomes: data from the TICOPA trial	See US table
Mathew et al. (66)	*Clin Rheumatol*. (2018)	Magnetic resonance imaging (MRI) of feet demonstrates subclinical inflammatory joint disease in cutaneous psoriasis patients without clinical arthritis	Evidence of inflammation was present in 33.9 and 50% patients in the PsO and PsA groups, respectively. Early arthritis for psoriatic patients screening questionnaire (EARP) score of ≥ 3 was significantly associated with imaging features of inflammation in PsO group (*p* = 0.044). The study suggested a high proportion of subclinical inflammation in small joints of foot in PsO patients.
Faustini (67)	*Ann Rheum Dis*. (2016)	Subclinical joint inflammation in patients with psoriasis without concomitant psoriatic arthritis: a cross-sectional and longitudinal analysis.	Prevalence of subclinical inflammatory lesions is high in patients with cutaneous psoriasis. Arthralgia in conjunction with MRI synovitis constitutes a high-risk constellation for the development of PsA.
**MRI and axial involvement in PsA**			
Maksymowych et al. (69)	*Ann Rheum Dis*. (2019)	MRI lesions in the sacroiliac joints of patients with spondyloarthritis: an update of definitions and validation by the ASAS MRI working group	Multi-reader validation demonstrated substantial reliability for the most frequently detected lesions and comparable reliability between active and structural lesions
Monaldo-Ficco et al. (70)	*Musculoskelet Dis*. (2017)	Magnetic Resonance Imaging in Psoriatic Arthritis: A Descriptive Study of Indications, Features and Effect on Treatment Change.	Magnetic resonance imaging is useful in evaluating patients with active PsA, particularly when suspecting inflammation and radiographic findings are unhelpful. In some cases, it can be used as an adjunct to clinical examination in determining treatment change.
Poggenborg et al. (71)	*Rheumatol Oxf Engl*. (2015)	Head-to-toe whole-body MRI in psoriatic arthritis, axial spondyloarthritis and healthy subjects: first steps toward global inflammation and damage scores of peripheral and axial joints	Whole-body MRI (WBMRI) allows simultaneous assessment of peripheral and axial joints in PsA and SpA, and the distribution of inflammatory and structural lesions and global scores can be determined. The study strongly encourages further development and longitudinal testing of WBMRI techniques and assessment methods in PsA and SpA

## Novel Techniques

Bone lesions can be divided into erosive or secondary to new bone formation. HRpQ-CT is a novel and promising technique that allows for the study of both, and it is mainly used in the diagnosis and evaluation of the disease progression (72–74). Simon et al. (74) evaluate the joints of 203 individuals affected by PSO, PsA, or healthy subjects. The presence of erosions in the PsA group was higher than in the control groups (140 erosions found vs. 27 in PSO group or in healthy controls; *p* = 0.002 for both comparisons). The volume of erosions was also significant with greater values in patients affected by PsA vs. PSO and healthy controls (*p* = 0.003 and 0.004, respectively). Nonsubstantial differences were found between PSO and healthy controls, and the conclusions of the authors are about a higher grade of involvement in PsA than in PSO or healthy status. This is due to an inflammatory trigger, which could lead to a heavier burden of the disease.

A similar work by Henchie et al. (75) tries to outline an image-based method for measuring joint deformities, considering erosions or bone growth. The algorithm is predictive and attempts to elaborate the prior healthy bone surface in a probabilistic manner. The authors considered the second metacarpal phalangeal joint of patients affected by PsA or RA. The algorithm was tested with computed models and surfaces altered by artificial erosions and bone growth in order to define the sensibility and specificity of the reconstructed model. For both PsA and RA, the number of lesions (erosions) was higher than that of the control group composed of healthy subjects. Even if the authors stated limitations of the algorithm, the method is still able to discriminate the volume and number of erosions, confirming the data already present in the literature. In addition, its development might lead to the creation of a valuable tool for the diagnosis and reconstruction surgery of the hand.

Only one study from Kampylafka et al. (36) reports data on HRpQ-CT variations before and after therapy. In patients treated with secukinumab, no progression is shown for enthesophytes or erosions after 24 weeks [erosions: median at baseline 2 (IQR = 0.5–4.5) and 2 (IQR = 1–4) after 24 weeks; entesophytes from 2 (IQR = 1–2) to 2 (IQR = 1.5–2)].

Another novel technique is DECT. It allows the acquisition of two different sets of images using different X-ray levels of energy. Two papers from Fukuda et al. (76, 77) were published in 2017 and focused on the diagnostic advantages of the technique. The first paper is about a prospective study that evaluated 16 patients, scoring the damage using the PsA MRI scoring system (PsAMRIS) (54) and comparing DECT with MRI. The sensitivity and specificity were 78 and 87%, respectively. The agreement with MRI was 86.3%. The authors found that DECT more often detects inflammatory lesions of the distal interphalangeal joints (DIP) or proximal interphalangeal joints (PIP). More in general, according to the authors, DECT supports inflammatory findings more often than MRI, but it is not found in metacarpal phalangeal joints (MCP) because this region is more extended in comparison with DIP and PIP. MRI is less limited in studying the surface area covered by DECT. The second paper speculates on the potential usefulness of DECT using iodine mapping for the study of peripheral arthritis. The comparison of synovitis highlights differences between RA and PsA with a prominent presence of inflammatory lesions in extra-articular structures, such as pulleys. The technique also seems to be useful in studying typical features, such as paratenonitis and ligaments. This study by Fukuda et al. (77) suggests that DECT iodine mapping could be a reliable technique that combines many advantages for diagnosis, assessment of damage, and drug response.

One final promising technique is fluorescence optical imaging (FOI). FOI is based on light-emitting diodes in the near-infrared spectrum that excite the fluorescence of an intravenously applied dye indocyanine green (ICG), which is used as contrast enhancer (78). In this manner, it can detect pathologic vascular changes, especially in areas such as the nail-enthesis complex.

Currently, there are only few studies investigating FOI in PsA. In 2019, Wiemann et al. (78) analyzed the characteristics of FOI signals in a cohort of PsA patients and found one particular pattern (“green nail”) to be highly specific (97%) for PsA.

Another study investigated the role of FOI in the early diagnosis of PsA in a smaller group of patients with certain vs. uncertain PsA and compared FOI with US (79). FOI seemed to be slightly more sensitive than US in detecting very early signs of PsA with the advantage of a comprehensive overview of the hand's structures. The main technical limitations are the need of ICG, that needs to be intravenously administrated and, at the current state of development, the fact that only hands and wrists can be examined.

## Conclusions

Imaging is becoming more important with time. It is probably the best “clinical biomarker” for spondyloarthritis because laboratory tests are poor in specificity and not satisfactory in detailing disease activity. In past decades, the canonical division between conventional (standing for XR) and advanced (US and MRI) has been widely adopted. Currently, this classification is no longer valid. The so-called advanced techniques became more available; thus, the old distinction is now based on several peculiarities of the imaging tool used. XR remains the standard for the therapy response and is a signature mark for every study that takes into account outcomes of disease progression. US and MRI are very useful for assessing early signs of PsA activity with a comprehensive evaluation of almost all structures involved. Promising data, especially in the field of CT, are now emerging (Table 3). The opportunity to obtain data for both anatomical and functional evaluation makes DECT very promising in this field. Furthermore, a novel approach to PsA damage might be given by HRpQ-CT because new bone formation and erosions are clearly seen even in the very early stage of the disease.

**Table 3:** Features and perspectives of the different imaging techniques in PsA.

**Imaging technique**	**Strengths**	**Limitations**	**Recommendations in clinical practice**	**Future perspectives**
X-Rays	Widely available, cost effectiveness, quick execution, viable for both peripheral and axial structures	Ionizing radiations, 2D imaging, low sensitivity, unable to provide information on disease activity	Recommended for diagnostic and follow up purposes	Longitudinal long-term data on structural disease progression in axial disease
Ultrasonography	Increasingly available, possibility of bedside examination, quick execution, good morphological detail (for accessible structures), provides information on disease activity, no ionizing radiations	Requires adequate training, currently useless for axial lesions	Recommended for diagnostic and follow up purposes	Longitudinal long-term data on structural disease progression in peripheral disease, new data on nail US
MRI	Very good morphological detail, useful for both axial and peripheral structures, no ionizing radiations, provides information on disease activity	Expensive, time-consuming, not widely available, requires contrast-enhancement (especially for peripheral structures)	Recommended for diagnostic and follow up purposes (recommendation still debated for axial involvement)	Longitudinal long-term data on structural disease progression and disease activity in peripheral and axial disease, further characterization of MRI lesions (i.e. fatty lesions, BME)
CT	Optimal morphological detail, reasonably quick execution	Requires ionizing radiations, no information on disease activity	Recommended in selected cases for diagnostic and/or follow up purposes limitedly for the assessment of structural damage	Longitudinal long-term data on structural disease progression and disease activity in peripheral and axial disease
DECT			N.A.	
HR-pQCT	Extreme structural detail	Requires ionizing radiations, no information on disease activity, viable only for research purposes, lack of currently validated definitions for elementary lesions in PsA	N.A.	Ultra-structural characterization of elementary peripheral lesions, longitudinal data on drug induced modification of bone microstructure, validation of elementary lesions, follow up and quantification of bone pathologic neoformation (enthesophytes)

*MRI, magnetic resonance imaging; CT, computed tomography; DECT, dual energy computed tomography; HR-pQCT, high-resolution peripheral quantitative computed tomography; US, ultrasonography; BME, bone marrow edema; PsA, psoriatic arthritis*.

## Author Contributions

All authors listed have made a substantial, direct and intellectual contribution to the work, and approved it for publication.

## Conflict of Interest

AF reports personal fees from Abiogen, Novartis, Neopharmed, outside the submitted work. LI reports personal fees from Eli-Lilly, Merck Sharp and Dohme, Novartis, Sanofi, Celgene, UCB outside the submitted work. The remaining author declare that the research was conducted in the absence of any commercial or financial relationships that could be construed as a potential conflict of interest.
